# miR-205 inhibits the development of hypertrophic scars by targeting THBS1

**DOI:** 10.18632/aging.104044

**Published:** 2020-11-13

**Authors:** Dongwen Jiang, Bingyu Guo, Feng Lin, Shixiu Lin, Kai Tao

**Affiliations:** 1 Reconstructive and Plastic Surgery, General Hospital of Northern Theater Command, Shenyang, P.R.China; 2 Graduate School, Jinzhou Medical University, Jinzhou 121001, P.R.China

**Keywords:** miR-205, THBS1, hypertrophic scars, growth, migration

## Abstract

Increasing evidence shows that miRNAs are involved in the growth and development of hypertrophic scars. However, the specific mechanism of miR-205 is unclear. Here, we investigated the relationship between miR-205, thrombospondin 1 (THBS1) expression, and hypertrophic scars, and showed that miR-205 inhibits cell proliferation and migration and induces apoptosis. Double luciferase analysis, Western blot, and real-time polymerase chain reaction showed that miR-205 downregulates THBS1 expression and activity. Compared to the control group, miR-205 inhibited hypertrophic scar development. Our findings contribute to a better understanding of the miR-205-THBS1 pathway as a promising therapeutic target for reducing hypertrophic scars.

## INTRODUCTION

Hypertrophic scars can be caused by deep burns, severe trauma, or abnormal repair of the skin after infection [1]. They are characterized by abnormal proliferation of fibroblasts in the dermis and excessive deposition of extracellular matrix. Hypertrophic scar contracture and excessive proliferation can lead to a change of local appearance and dysfunction of surrounding organs and joints, resulting in immense pain [2, 3]. Although numerous scar treatments are currently available, many options have significant limitations and side effects. Further, it is difficult to obtain the ideal effect with a single method [4]. Therefore, revealing the pathological mechanism of hypertrophic scars could provide a theoretical basis for seeking an effective treatment.

MicroRNA (miRNA) interaction with target genes is complex. miRNAs play important roles in regulating biological cell growth and development, proliferation, differentiation, maturation, and apoptosis. Moreover, abnormal expression of miRNAs has previously been observed in hypertrophic scars [5]. miR-222 has been shown to target MMP-1, regulating the proliferation and migration of hypertrophic scar fibroblasts [6]. Additionally, miR-486-5p has been reported to inhibit proliferation and collagen production of hypertrophic scar fibroblasts through the IGF1/PI3K/Akt pathway [7]. Previous studies have shown that miR-145-5p reduces Smad2/Smad3 expression in hypertrophic scars [5], and that miR-29b inhibits the development of hypertrophic scars by regulating the TGF-β1/Smad signal pathway [8]. miR-205-5p has also been shown to regulate extracellular matrix production in hyperplastic scars by targeting Smad2 [9].

Thrombospondin-1 (THBS1) is an extracellular glycoprotein secreted by platelet Q granules, fibroblasts, and vascular endothelial cells. THBS1 is involved in embryo development, neovascularization, tissue repair, and other important physiological processes that are closely related to the occurrence and development of many diseases, especially tumors [10]. More attention, however, has recently been given to the role of THBS1 in fibrotic diseases [11]. Transforming growth factor beta-1 (TGF-β1) is a multifunctional cytokine, which plays a key role in wound healing and tissue repair, and is a central mediator that contributes to skin fibrosis [10]. THBS1 plays an important role in the activation of potential TGF-β1 [12]. It can also protect TGF-β1 from extracellular inactivator influence allowing TGF-β1 activity to persist in the LAP-TSPI-TGF-β complex [13]. Upon activation, TGF-β1 binds to the corresponding type I and type II receptors initiating signal transduction that plays a role in TGF-β1–induced fibrosis [12]. However, few studies have been conducted on the regulatory effect of THBS1 on hypertrophic scars.

In this study, we examined the expression, biological function, and potential mechanism of miR-205 in hypertrophic scars and show how miR-205 affects hypertrophic scars by targeting THBS1.

## RESULTS

### Downregulation of miR-205 expression in hypertrophic scars

**Figure 1:**
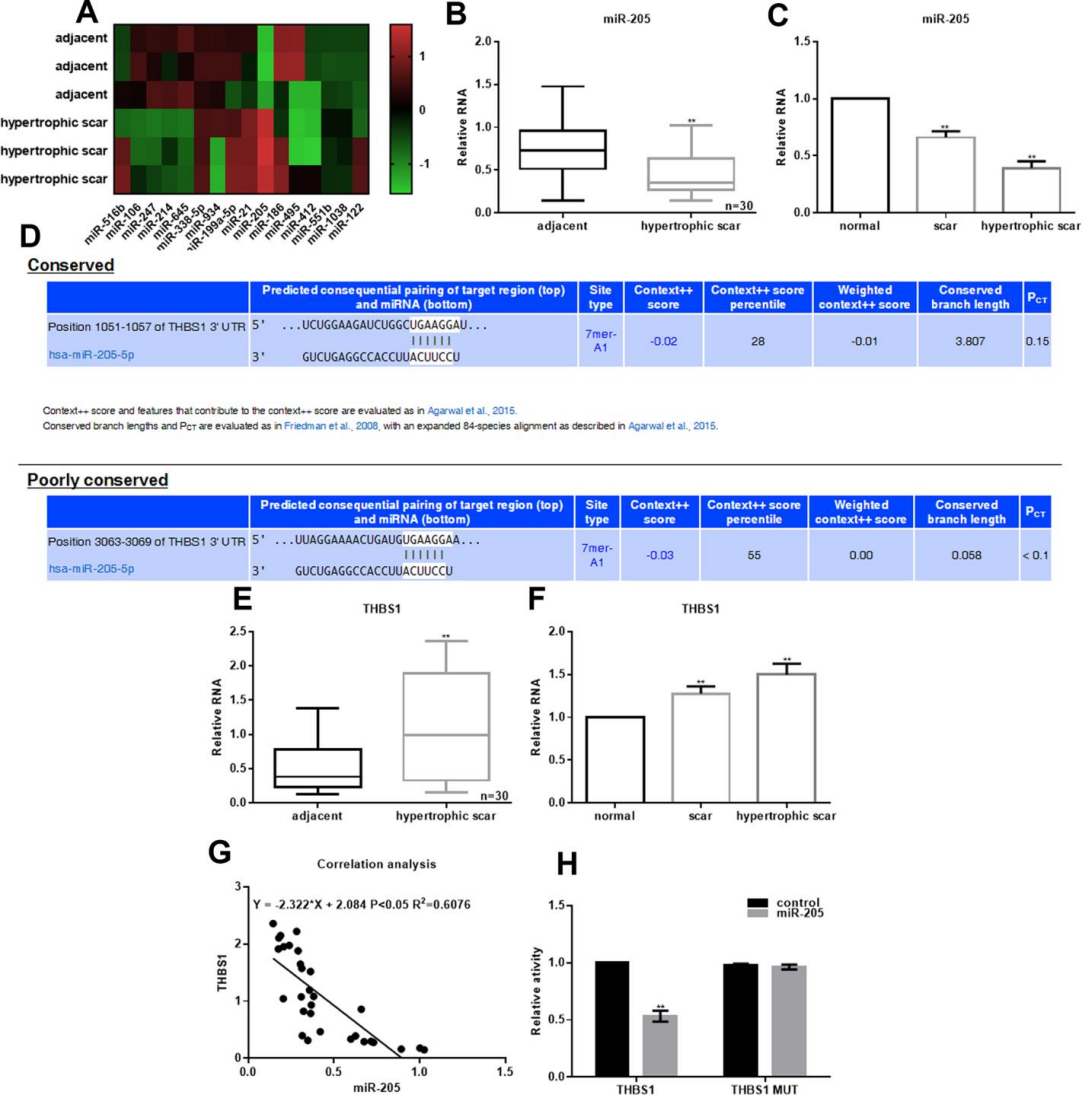
**Downregulation of miR-205 expression in hypertrophic scars.** (**A**) Different expression of miRNAs in adjacent skin and hypertrophic scars was accessed by miRNA microarray analysis. (**B**, **C**) miR-205 expression in hypertrophic scars tissues and fibroblasts was examined. **P < 0.05. (**D**) The schematic of binding sites between miR-205 and THBS1 3’-UTR. (**E**, **F**) THBS1 expression in hypertrophic scars tissues and fibroblasts was examined. **P < 0.05. (**G**) The correlation between miR-205 and THBS1 expression was estimated. *P* < 0.05 was considered statistically significant. (**H**) The relative dual-luciferase activity of THBS1 in cells expressing miR-205 was determined by dual-luciferase assay. **P < 0.05, *vs.* control.

To investigate the expression of miRNAs between adjacent skin and hypertrophic scars, PCR-based microarrays were performed. Figure 1A shows the most significant changes in miRNA expression. miR-205 expression was also examined in 30 cases of hypertrophic scars and cells. Figure 1B shows that miR-205 expression was lower in hypertrophic scars than in the adjacent tissues. miR-205 expression was also lower in hypertrophic scar cells compared with the adjacent and scar tissues (Figure 1C). Moreover, Figure 1D shows that miR-205 directly binds with THBS13 '- UTR (TargetScan database). These results showed that THBS1 expression in hypertrophic scar tissue and cells was significantly upregulated (Figure 1E, 1F). There was a negative correlation between miR-205 expression and THBS1 in hypertrophic scars (Figure 1G). In the cells expressing miR-205, relative dual-luciferase activity of THBS1 wild-type promoter decreased about 40%, whereas in the cells expressing THBS1 mutant, there was almost no change (Figure 1H).

### miR-205 inhibits growth and migration of hypertrophic scar fibroblasts

**Figure 2:**
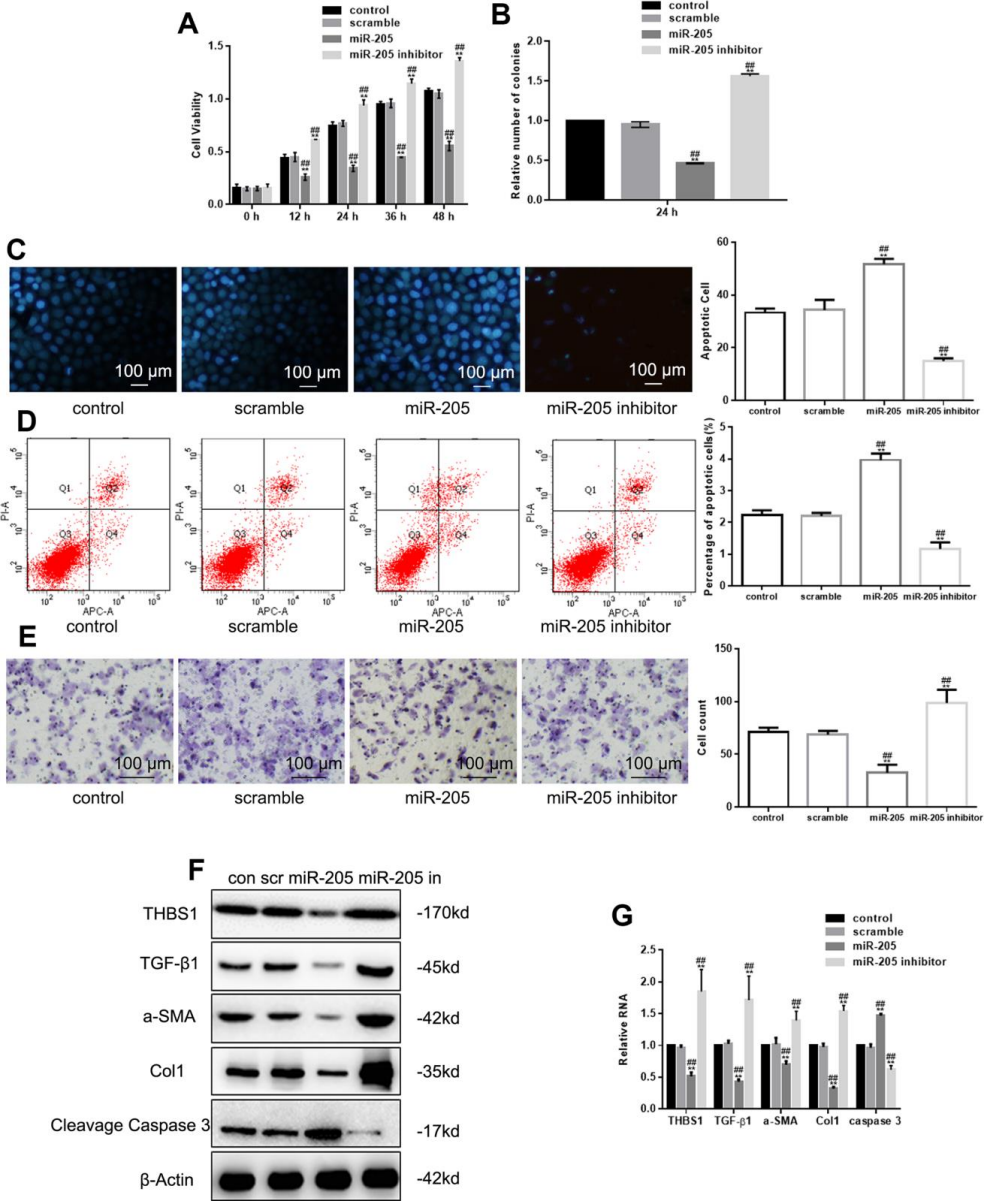
**miR-205 inhibits growth and migration of hypertrophic scar fibroblasts.** (**A**, **B**) The viability of cells deprived miR-205 or expressing miR-205 was measured by MTT and colony formation assay. **P < 0.05, vs. control. ##P < 0.05, vs. scramble. (**C**, **D**) The apoptosis of cells deprived miR-205 or expressing miR-205 was measured by Hoechst 33258 assay and Annexin V-PI assay. **P < 0.05, vs. control. ##P < 0.05, vs. scramble. (**E**) The migration of cells deprived miR-205 or expressing miR-205 was measured by Transwell assay. **P < 0.05, vs. control. ##P < 0.05, vs. scramble. Results show the mean ± SD of three independent experiments. (**F**, **G**) THBS1, TGF-β1, α-SMA, Col1, and Cleavage Caspase 3 expression was detected. Cells were transfected with control, scramble, miR-205, and miR-205 inhibitor, respectively. **P < 0.05, vs. control.

MTT assay and colony formation assay indicated that miR-205 overexpression hampered cell growth, whereas miR-205 deregulation promoted growth (Figure 2A, 2B). In addition, Hoechst 33258 and Annexin V-PI assay showed that apoptotic cells decreased in miR-205–deficient cells, but increased in miR-205–overexpressed cells (Figure 2C, 2D). In addition, miR-205 inhibition decreased cell migration regulation (Figure 2E). Similar to the important roles that TGF-β1, α-SMA, and Col1 play in hypertrophic scarring, Cleavage Caspase 3 is an important indicator of apoptosis. We also studied the effect of miR-205 on α-SMA, Col1, and TGF-β1 expression. Western blot and real-time PCR showed that miR-205 downregulated THBS1, TGF-β1, α-SMA, and Col1 expression, and upregulated Cleavage Caspase 3 (Figure 2F, 2G).

### miR-205 restores the effect of THBS1 overexpression on cells

**Figure 3:**
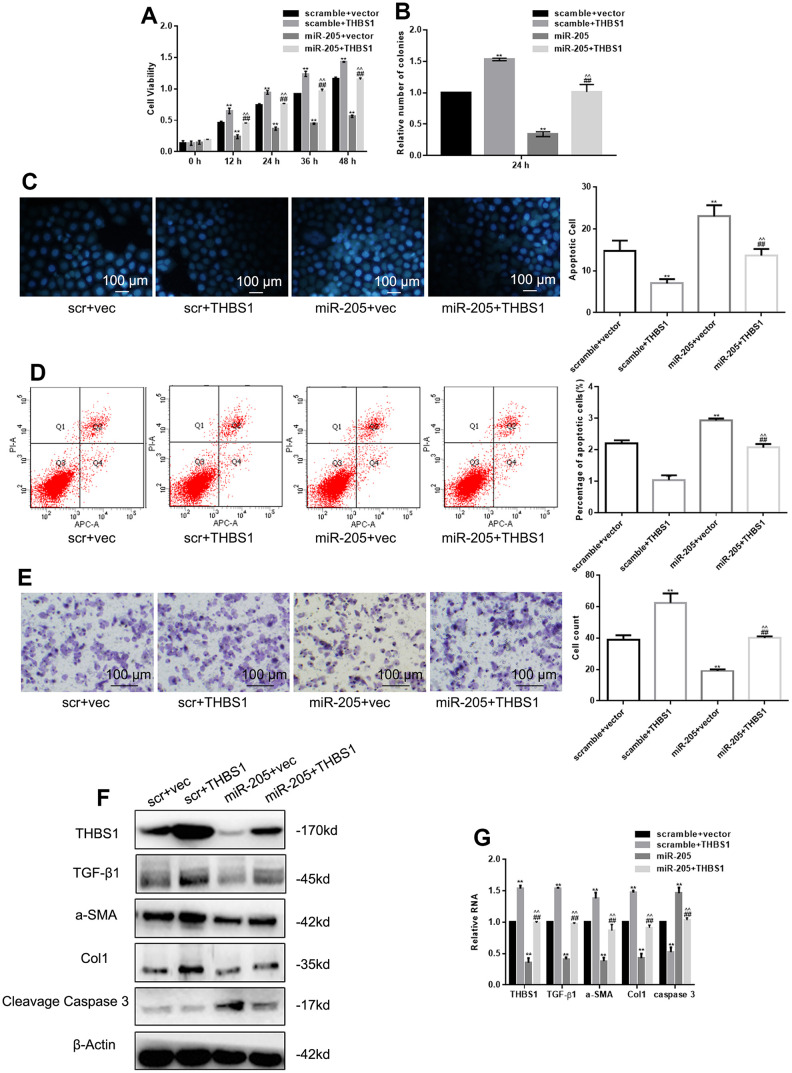
**miR-205 restored the effect of THBS1 overexpression on cells.** (**A**, **B**) The viability of the indicated cells was detected by MTT assay and colony formation assay. **P < 0.05, vs. scramble+vector. ##P < 0.05, vs. scramble+THBS1. ^^P<0.05, vs. miR-205+vector (**C**, **D**) The apoptosis of the indicated cells was detected by Hoechst 33258 and Annexin V-PI assay. **P < 0.05, vs. scramble+vector. ##P < 0.05, vs. scramble+THBS1. ^^P<0.05, vs. miR-205+vector (**E**) The indicated cell migration was measured by Transwell assay. **P < 0.05, vs. scramble+vector. ##P < 0.05, vs. scramble+THBS1. ^^P<0.05, vs. miR-205+vector (**F**, **G**) Expression of the indicated genes was measured by Western blot and real-time PCR, respectively. The cells were transfected with scramble plus vector, scramble plus THBS1, miR-205 plus vector, and miR-205 plus THBS1. Results show the mean ± SD of three independent experiments.

MTT assay, colony formation assay, Hoechst 33258, Annexin V-PI assay, and Transwell assay were used to detect the proliferation, apoptosis, and migration of hypertrophic scar cells. As shown in Figure 3A, 3B, THBS1 overexpression promoted cell proliferation and miR-205 overexpression inhibited cell proliferation, whereas THBS1 restored the inhibitory effect of mir-205 on cell proliferation. In addition, miR-205 plus THBS1 overcame the increase of apoptosis induced by miR-205 (Figure 3C, 3D). Notably, the inhibition of miR-205 on cell migration resumed after THBS1 overexpression (Figure 3E). The detection of protein and RNA levels indicated that THBS1 restored related protein expression downregulation caused by miR-205 (Figure 3F, 3G).

### miR-205 inhibitor restores the effect of si-THBS1 overexpression on cells

**Figure 4:**
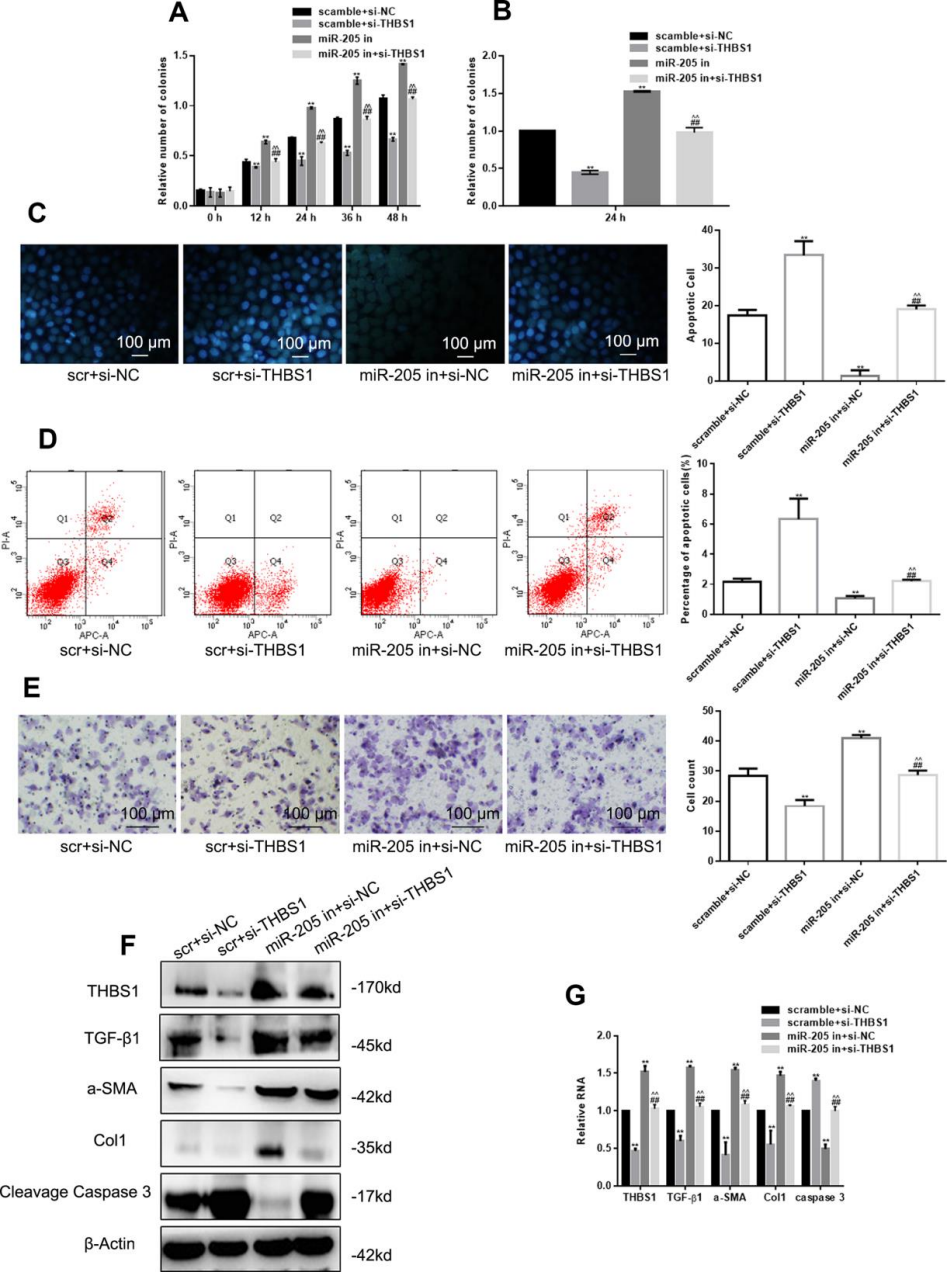
**miR-205 inhibitor can restore the effect of si-THBS1 overexpression on cells.** (**A**, **B**) The viability of the indicated cells was detected by MTT assay and colony formation assay. (**C**, **D**) The apoptosis of the indicated cells was detected by Hoechst 33258 and Annexin V-PI assay. (**E**) The indicated cell migration was measured by Transwell assay. (**F**, **G**) The expression of the indicated genes was measured by Western blot and real-time PCR, respectively. **P < 0.05, vs. scramble+si-NC. ##P < 0.05, vs. scramble+si-THBS1. ^^P<0.05, vs. miR-205+ si-NC The cells were transfected with scramble plus si-NC, scramble plus si-THBS1, miR-205 inhibitor plus si-NC, and miR-205 inhibitor plus si-THBS1. Results show the mean ± SD of three independent experiments.

MTT assay and colony formation showed that THBS1 silencing inhibited cell proliferation seriously (Figure 4A, 4B). miR-205 downregulation restored the inhibition of cell proliferation induced by THBS1 silencing. Hoechst 33258 and Annexin V-PI assay showed that miR-205 downregulation restored upregulation of THBS1-induced apoptosis (Figure 4C, 4D). Figure 4E shows that THBS1 silencing inhibited cell migration, and miR-205 upregulated cell migration. The detection of protein and RNA levels indicated that the function of miR-205 inhibitor could be restored by si-THBS1 (Figure 4F, 4G). The previous evidence shows that miR-205 can inhibit cell proliferation, migration, and promote apoptosis by targeting THBS1.

### miR-205 arrests hypertrophic scars in hypertrophic scar mice models

**Figure 5:**
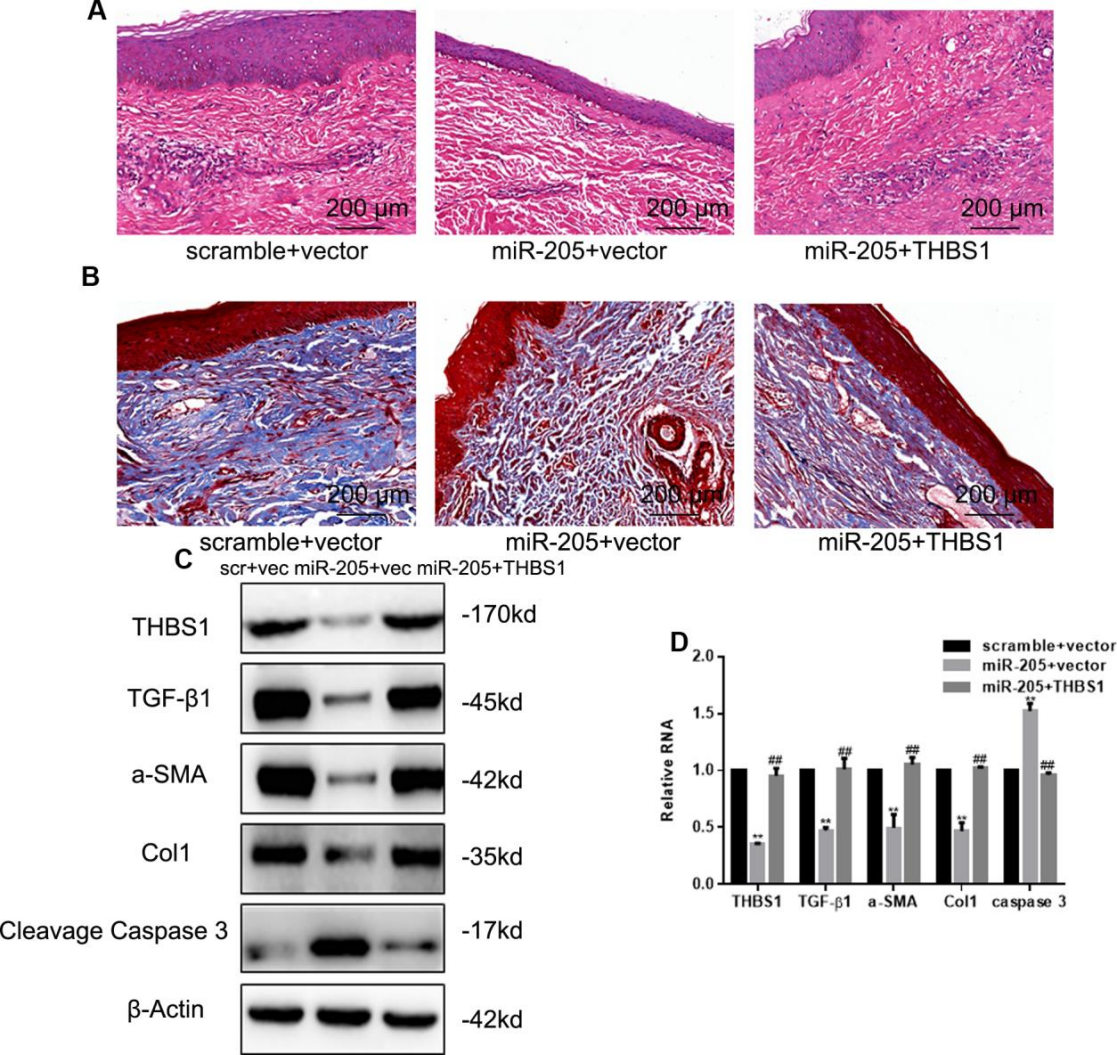
**miR-205 arrests hypertrophic scars in hypertrophic scar mice models.** (**A**) The representative images of the HE examination of hypertrophic scars are shown. (**B**) The representative images of the Masson assay examination of hypertrophic scars are shown. (**C**, **D**) THBS1, TGF-β1, α-SMA, Col1, and Cleavage Caspase 3 expression was accessed by Western blot and real-time PCR, **P < 0.05, *vs.* control. ##P < 0.05, vs. miR-205+vector.

To observe the effects of miR-205 on hypertrophic scars, a hypertrophic scar mice model was created by injecting mice with bleomycin, then injecting the mice with negative control, miR-205 or miR-205, and THBS1. Figure 5A, 5B shows that miR-205 hindered hypertrophic scar growth and collagen formation compared with the normal control (NC) group. miR-205 expression decreased THBS1, TGF-β1, α-SMA, and Col1 expression and upregulated Cleavage Caspase 3 expression (Figure 5C, 5D). Our results showed low expression of miR-205 in hypertrophic scar tissue, and that miR-205 regulates the occurrence and development of hypertrophic scar by regulating THBS1 (Figure 5E).

## DISCUSSION

miRNA has recently become a “hot spot” in the study of pathological mechanism of various diseases. Further evidence reveals the important role miRNA has in the potential treatment of chronic and future fibrotic diseases [14]. In the process of wound healing, fibroblasts are regulated by a variety of cytokines [15]. When activated, they can synthesize and secrete a large number of extracellular matrix and cytokines, such as collagen, and elastic fibers. The occurrence, development, and harm of hypertrophic scars depend on the biological characteristics of fibroblasts as well as cell status [16]. In-depth studies have shown that miRNA plays a role in cell differentiation, maturation, apoptosis, and proliferation. miRNA is closely related to the occurrence and development of various diseases in multiple systems. Further, miRNA has previously been shown to play a key role in abnormal skin wound healing and various skin diseases [2]. Therefore, to investigate the miRNA expression profile in hypertrophic scars and normal skin tissue, miR-495 was analyzed and shown to inhibit the growth of hypertrophic scar fibroblasts [17]. miR-137 was found to inhibit the proliferation and metastasis of hypertrophic scar fibroblasts by targeting polymerized proteins [18]. In their study, Wu et al. report that miR-155 targeted HIF-1α through the PI3K/Akt pathway, inhibiting formation of hypertrophic scar fibroblasts [19]. In a different study, the abnormal expression of miR-21 and miR-200b was shown to promote the potential of hypertrophic scar fibrosis [20]. miR-10a and miR-181c were also found to regulate the formation of type I collagen in hypertrophic scars by targeting PAI-1 and uPA [21]. Further, Xiao et al. reported that miR-564 promotes the formation of hypertrophic scars by upregulating TGF-β1 [22].

High-throughput Agilent human miRNA microarray was used to analyze and compare the miRNA molecules differentially expressed in 30 pairs of hypertrophic scar and normal skin tissues. Real-time PCR was then used. The microarray analysis results showed that miR-516b, miR-199a-5p, miR-21, miR-205, and miR-122 had abnormal expression in hypertrophic scars, of which miR-205 downregulated most significantly in hypertrophic scars, and miR-205 expression in normal tissues was about 7.4 times that in hypertrophic scars. In addition to the application of hypertrophic scar, normal scar, and normal skin tissue samples, the corresponding fibroblasts were also obtained in primary cultures of three kinds of tissue, and a quantitative real-time PCR (qRT-PCR) method was used to further verify the results of gene chip analysis. The microarray results showed that miR-205 expression in hypertrophic scar fibroblasts was significantly lower than that in normal tissues and scars.

A previous study reported that miR-205 expression downregulated in the fibrotic lung tissue of bleomycin-induced pulmonary fibrosis mice, and that miR-205 may play a role in inhibiting the pulmonary fibrosis of mice by targeting GATA3 [23]. miR-205-5p may play a key role in cell proliferation, apoptosis, and extracellular matrix deposition [24]. miR-205 upregulation has previously been shown to inhibit VEGF expression in human keloid fibroblasts through the PI3K/Akt signaling pathway [24]. miR-205-5p inhibits the production of extracellular matrix by targeting Smad2 [9]. To reveal the specific molecular mechanism of miR-205 in regulating the occurrence and development of hypertrophic scars, we predicted and analyzed the target gene of miR-205 through the database according to the basic principles of bioinformatics. According to the database screening, miR-205-5p interacts with multiple mRNA. Because of the classic role of TGF-β1 in hypertrophic scars [25], we selected THBS1, which can affect TGF-β1. In addition, the data indicated that miR-205-5p expression negatively correlated with THBS1 in 449 skin melanoma samples, and that miR-205 affects the proliferation and metastasis of melanoma cells by targeting CCL18 [26]. We conclude that miR-205-5p may play an important role in hypertrophic scars by regulating THBS1. The contents of type I collagen, α-SMA, and THBS1 in pathological scars were higher than those in normal fibroblasts [27]. THBS1 has been shown to play a role in regulating the biological process of pathological scars by affecting the fibroblasts, collagen, and some immune cells, which are located near the infiltration of immune cells [28]. THBS1 can activate potential TGF-β by binding to latent related peptide-β on the TGF-β structure. Selective inhibition of TSP-1 has been shown to inhibit the development of hypertrophic scars by PI3K/Akt/mTOR signal [13]. In our study, THBS1 upregulated in hypertrophic scars. Here, a targeted binding site was shown between THBS1 and miR-205, and that miR-205 significantly downregulated THBS1 expression. To further understand the biological function of miR-205 in hypertrophic scar fibroblasts, we first constructed hypertrophic scar fibroblasts with overexpression or low expression of miR-205, and detected the changes of cell proliferation, apoptosis, and migration ability. The proliferation level of hypertrophic scar fibroblasts in the control group was significantly higher than that in the miR-205 group. After miR-205 overexpression, the proliferation of primary fibroblasts was significantly inhibited. The apoptosis rate of fibroblasts in the miR-205 group was significantly higher than that in the control group, indicating that the low expression of miR-205 in hypertrophic scars may be an important reason for the over proliferation of fibroblasts. The Transwell experiment showed that miR-205 overexpression could inhibit the migration of fibroblasts in hypertrophic scars.

Our results further show that the regulatory effect of miR-205 on the biological function of hypertrophic scar fibroblasts are weakened by THBS1 transfection. In vivo experiments showed that miR-205 could inhibit hypertrophic scar formation and collagen synthesis.

In conclusion, by upregulating or inhibiting the expression of miR-205, this study confirms that miR-205 can affect hypertrophic scars by targeting THBS1 regulation. The current evidence provides new insight for development of hypertrophic scars, expands on the knowledge of complex miRNA-mRNA interaction in tumor metastasis, and promotes the development of diagnosis and treatment of hypertrophic scars.

## MATERIALS AND METHODS

### Patient recruitment and tissues sample collection

Tissue samples were provided by the Plastic Surgery Department of the General Hospital of Northern Theater Command. Hyperplastic scar specimens (30 cases total: 18 chest, 6 arm, and 6 leg; 14 male and 16 female participants, with an average age of 35.98 years ± 6.74) were preserved and selected after surgery. Normal scar tissue (6 cases total: 1 leg and 5 chest; 2 male and 4 female participants, with an average age of 37.92 years ± 8.77) and adjacent skin tissues (30 cases total: hyperplastic scar specimens) that were excised at the same time were selected as the control. Samples of patients who had received hormones, radiation, topical or local injection of any drug, as well as patients with any other skin, immune, or infectious diseases were excluded. Each sample collected was divided into two parts; one part was fixed and stored in paraffin, the other was frozen at –80°C. The study was approved by the Medical Ethics Committee of the General Hospital of Northern Theater Command.

### miRNA microarray analysis

Hypertrophic scars tissues were screened for miRNA using Agilent human miRNA V21.0 (Agilent Technologies Co. Ltd., Shanghai, China). miRNA array analysis was conducted by GeneStudy_1.0.030.1023, according to the manufacturer’s instructions. miRNA expression (fold change >1.5, *P*-value < 0.05) was considered statistically significant. Data from three independent experiments were analyzed.

### Cell culture

All specimens were obtained with written informed consent of patients. Hypertrophic scar, normal scar, and adjacent normal tissues were cut to sections, incubated with trypsin at 37°C for 1 hour, and then cultured in Dulbecco’s modified Eagle’s medium (DMEM; Invitrogen, Carlsbad, CA, USA) containing 10% fetal bovine serum. After 72 hours of attachment, a large number of fibroblasts could be seen under the microscope. The tissue blocks were removed and cultured for 2–3 days. When the cells were full, they could be passaged.

### Transfection

For each sample, a Lipofectamine2000 (lipo2000) mixture was prepared as follows: dilute miRNA control (scramble)/mimic/inhibitor (Life Technologies, Carlsbad, CA, USA): dilute with 50 μL of DMEM and 1.5 μL of a storage solution, then incubate at room temperature for 5 min; dilute liposome lipo2000: dilute 1 μL of lipo2000 with 50 μL of DMEM, incubate at room temperature for 5 min; mix, then incubate at room temperature for 20 min. The mixed solution was then added to the cells, and after 6 hours, the fresh medium was replaced. The RNA sequence is listed in Table 1.

**Table 1:** RNA sequences.

**Name**	**sequence**
miR-205 scramble	UUUGUACUACACAAAAGUACUG
miR-205 mimic	UCCUUCAUUCCACCGGAGUCUG
miR-205-5p inhibitor	CAGACUCCGGUGGAAUGAAGGA

### Prediction of miRNA: protein interactions

TargetScan online tool (http://www.targetscan.org/vert_71/) [29, 30] was used to estimate the miRNA target genes.

### MTT assay

Cell activity testing was based on MTT (3 - [4, 5-dimethylthiazole-2-yl] - 2,5-diphenyltetrazole ammonium bromide; Sigma-Aldrich, St. Louis, MO, USA), according to the product specifications. A total of 1x10^4^ cells were inoculated in 96-well plates and treated with different solutions. Cells were incubated with 10 μL of MTT solution (5 mg/mL) at 37°C for 4 hours. Then, 150 μL of DMSO was used to replace the medium, and the crystals were dissolved for 10 min. The optical density at 490 nm was read with a micro reader (Life Sciences, Hercules, CA, USA).

### Colony formation assay

Cells were plated in 6-well plate at 500 per well. After treatment with different solutions, the cells were cultured for 24 hours at 37°C, and colonies were washed with PBS, fixed, and stained with hematoxylin. Colonies with more than 50 cells were counted under a microscope.

### Hoechst 33258

After incubation at 37°C for 24 hours, Hoechst 33258 working solution was stained for 15 minutes at and observed using a fluorescence microscope (X200).

### Annexin V-PI assay

To assess the percentage of apoptotic cells, the Annexin V FITC Apoptosis Detection Kit (BD Biosciences, San Jose, CA, USA) was used according to the manufacturer’s instructions.

### Migration assay

The modified solution (Sigma-Aldrich) was used for oral analysis. After treatment with different solutions, a total of 5 × 104 cells were inoculated in 0.2 mL of serum-free DMEM, located in the upper chamber of each cell chamber, whereas the lower chamber was filled with 0.6 mL of DMEM, and 10% FBS was added. After incubation at 37°C for 24 hours, the lower cells were stained with crystal violet solution (1% w/v) and counted under a microscope.

### Western blots

Thirty micrograms of protein were dissolved in SDS-PAGE gel, then transferred onto polyvinylidene difluoride membranes (Sigma-Aldrich) by electroblotting. After electroblotting, the membranes were blocked for 1 hour at room temperature with 5% blocking solution, the required bands were cut according to marker, and THBS1 antibodies (1:1000, Cell Signaling Technology, Cat# 37879), TGF-β1 (1:1000, Abcam, ab92486), α-SMA (1:500, Abcam, ab5694), Collagen I (Col-1, 1:1000, Abcam, ab6308), Cleavage Caspase 3 (1:1000, Cell Signaling Technology, Cat# 9661), and β-Actin (1:1000, Abcam, ab8227) were incubated overnight at 4°C. The second antibody (1:5000) was incubated at room temperature for 1 hour. Immunoreactivity was measured using the Western Lighting Ultra Kit (ECL, Pierce Technology, Shanghai, China).

### qRT-PCR

Centrifugation was performed at 12,000 rpm for 10 min at 4°C. A total of 200 μL of anhydrous ethanol was added, mixed well, and centrifuged at 8000 rpm for 1 min at 4°C. A total of 500 μL of RWA buffer was added and centrifuged at 12,000 rpm for 1 min at 4°C. Centrifugation was performed again at 12,000 rpm for 1 min at 4°C, the mixture was air-dried for 2 min, adding 50 μL of DEPC water, at room temperature for 2 min. RNA was collected by centrifugation at 12,000 rpm for 1 min at 4°C. One milligram of RNA was reverse transcribed to cDNA in 20-μL system by real-time reaction kit (Promega, Beijing, China). Real-time PCR was performed using the Mx3000P real-time PCR system (Applied Biosystems, Beijing, China). PCR was carried out as follows: 40 cycles of 94°C for 15 sec, 60°C for 10 sec, and 72°C for 20 sec. All procedures were repeated three times. Table 2 shows the primer sequences used in this study.

**Table 2:** RNA primers.

**Name**	**Forward primer (5'->3')**	**Reverse primer (5'->3')**
miR-205	GCTCCTTCATTCCACCGG	CAGTGCAGGGTCCGAGGT
U6	CTGCTTCGGCAGCACA	AACGCTTCACGAATTTGCGT
THBS1	GTCATACAACACTCCCACGC	CCAGGGCATAGGTAGAAGCT
TGF-β1	ATGCCGCCCTCCGGGC	TCAGCTGCACTTGCAGGAGCG
α-SMA	GGAAGGACCTCTATGCTAAC	CATAGGTAACGAGTCAGAGC
Col1	CGTGGAAACCTGATGTATGCT	ACTCCTATGACTTCTGCGTCTG
Caspase3	TGTGAGGCGGTTGTAGAAGTT	GCTGCATCGACATCTGTACC
GAPDH	AGCCACATCGCTCAGACAC	GCCCAATACGACCAAATCC

### Luciferase reporter assay

The miR-205 targeted gene expression was measured by using a dual-luciferase reporter assay in cells. The putative miR-205-5p complementary site in the 3’-UTR of THBS1 or its mutant sequence was cloned into the pmiR-RB-REPORT vector (RiboBio Inc.). Then, pmiR-RB-REPORT-THBS1-3’UTR-WT or pmiR-RB-REPORT-THBS1-3’UTR-MT were co-transfected into cells with miR-205-5p mimic or its negative control in 48-well plates, collected 48 hours after transfection, and analysed using a Dual-Luciferase Reporter Assay System (Promega). The Firefly luciferase signal was used for normalization.

### Animal models and drug administration

Male 6-week-old BALB/c mice received daily dorsal subcutaneous injections with bleomycin (100 μg/100 μL, dissolved in PBS) (n=12). Two weeks later, scramble (2 mg/kg)+vector (4 mg/kg), miR-205 (2 mg/kg)+vector (4 mg/kg) or miR-205 (2 mg/kg)+THBS1 (4 mg/kg) was injected subcutaneously daily [31]. Each mixture in 50 μL of DMEM was mixed with 2 μL of cationic liposomes [32]. After 3 weeks, mice were sacrificed and skin tissues were processed for analysis [33].

### Hematoxylin and eosin staining

The sections were dewaxed with gradient xylene solution, dehydrated with gradient ethanol solution, then washed with distilled water for 30 min, stained with hematoxylin for 2 min, washed with 1% hydrochloric acid ethanol for 5 sec, washed with water for 30 sec, stained with 0.5% eosin for 3 min, dehydrated with gradient ethanol, xylene transparent, and sealed with neutral gum.

### Masson staining

Paraffin section was dewaxed prior to staining and rinsed with water. The treatment consisted of: hematoxylin staining for 2 min; hydrochloric acid ethanol differentiation then rinsed with water; Ponceau acid red complex solution staining for 10 min; 2% glacial acetic acid solution immersion for 20 s; 1% phosphomolybdic acid solution differentiation for 3 min; aniline blue or bright-green solution staining for 5 min; 0.2% glacial acetic acid immersion for a moment; gradient ethanol dehydration, xylene transparent, and neutral gum sealing.

### Statistical analysis

Data from all experiments are shown as mean ± SD. The association of THBS1 and miR-205 expression was analyzed by Spearman’s correlation coefficient. The differences were evaluated by one-way analysis of variance with a least significance difference test. A *P* value of less than 0.05 was considered statistically significant. Statistical analysis was performed using GraphPad prism software version 7.1 (San Diego, CA, USA).
